# Molecular Tools for Monitoring *Trichoderma* in Agricultural Environments

**DOI:** 10.3389/fmicb.2018.01599

**Published:** 2018-07-25

**Authors:** László Kredics, Liqiong Chen, Orsolya Kedves, Rita Büchner, Lóránt Hatvani, Henrietta Allaga, Viktor D. Nagy, Jamal M. Khaled, Naiyf S. Alharbi, Csaba Vágvölgyi

**Affiliations:** ^1^Department of Microbiology, Faculty of Science and Informatics, University of Szeged, Szeged, Hungary; ^2^Department of Botany and Microbiology, College of Science, King Saud University, Riyadh, Saudi Arabia

**Keywords:** biocontrol agent, fingerprinting, marker, monitoring, PCR, specific detection, transformation, *Trichoderma*

## Abstract

Various *Trichoderma* species possess significance in agricultural systems as biofertilizers or biocontrol agents (BCAs). Besides these beneficial features, certain *Trichoderma* species can also act as agricultural pests, causing the green mold disease of cultivated mushrooms. This double-faced nature of the genus in agricultural environments points at the importance of proper monitoring tools, which can be used to follow the presence and performance of candidate as well as patented and/or registered biocontrol strains, to assess the possible risks arising from their application, but also to track harmful, unwanted *Trichoderma* species like the green molds in mushroom growing facilities. The objective of this review is to discuss the molecular tools available for the species- and strain-specific monitoring of *Trichoderma*, ranging from immunological approaches and fingerprinting tools to exogenous markers, specific primers used in polymerase chain reaction (PCR) as well as “omics” approaches.

## Introduction

Members of the filamentous fungal genus *Trichoderma* commonly occur in the soil and the rhizosphere of various plants. The genus has been a popular subject of basic and applied mycology research since the 1930s (Weindling, 1932), which is mainly due to the fact that *Trichoderma* species play important roles in various agricultural environments ranging from field and forest soil ecosystems to substrate materials used for mushroom production. Several members of the genus have the potential to control plant pathogenic fungi and nematodes by antagonistic action based on competition, antibiosis and/or parasitism, furthermore, the biostimulant ability of certain *Trichoderma* species enables to enhance the uptake of nutrients by plants, promote plant growth, increase crop productivity and induce systemic resistance in plants, which can also be exploited within the frames of environmentally friendly agricultural practices (Harman et al., 2004; Nawrocka and Małolepsza, 2013; Gupta et al., 2014; López-Bucio et al., 2015; Contreras-Cornejo et al, 2016). The original view considering *Trichoderma* species as biocontrol agents has recently evolved to the concept that they are avirulent, endophytic plant symbionts capable of long-lasting colonization and penetration of roots and providing the plants with various beneficial effects (Harman et al., 2004; Lorito et al., 2010). However, besides the positive implications of the genus, *Trichoderma* species may also be harmful for agriculture, like in the case of mushroom production, where *Trichoderma* occurs as the causal agent of green mold disease severely affecting cultivated mushrooms (Hatvani et al., 2008, 2017; Kredics et al., 2010). Moreover, certain species of the genus are known as opportunistic human pathogens, and the causal agents of different diseases may also originate from agricultural environments (Druzhinina et al., 2008).

Ecological fitness is an important trait of any *Trichoderma* strain to establish in agricultural habitats like soil, plant rhizosphere or compost materials (Weaver et al., 2005; Cordier et al., 2007). The survival and spread of *Trichoderma* in an agricultural habitat depend on its interactions with the environmental parameters as well as the biotic and abiotic components of the local ecosystem (Kredics et al., 2003). An increased knowledge about the inoculum source, survival, spread and general population dynamics of harmful *Trichoderma* species in the substrates of mushroom production may enable the development of more efficient control measures to avoid green mould outbreaks in mushroom farms. On the other hand, during the development of a beneficial *Trichoderma* strain to a BCA, a crucial step is the collection of basic information about its abilities to colonize, persist, and spread under the conditions characteristic to the environment where it will be applied (Longa et al., 2009). Monitoring the fate, behavior and population dynamics of *Trichoderma* strains released as BCAs into the environment is therefore of special importance. In Europe, the registration of new, *Trichoderma*-based biocontrol products requires the use of specific monitoring tools capable of accurately identifying and quantifying the released strain and tracking its population dynamics over time (Savazzini et al., 2008). Therefore, it is a challenge of increasing importance to differentiate the newly introduced *Trichoderma* strains from the natural *Trichoderma* populations occurring in an agricultural habitat (Dodd et al., 2004b).

The traditional microbiological approach in *Trichoderma* population studies is the use of *Trichoderma*-selective or semi-selective agar media (Elad et al., 1981; Chung and Hoitink, 1990; Williams et al., 2003; Vargas Gil et al., 2009) for dilution plating of environmental samples, which is followed by the calculation of colony forming units (CFU). However, only the total amount of *Trichoderma* propagules can be assessed by this technique, it does not enable the distinction of an introduced strain from the *Trichoderma* populations resident in the investigated environment. Neither morphological, nor nutritional criteria are suitable markers to reliably verify the identity of the colonies (Hermosa et al., 2001), and for the large number of samples examined during population studies, a colony-by-colony barcoding based on the sequence analysis of the ITS (internal transcribed spacer) region (Druzhinina et al., 2005) or a part of the translation elongation factor 1α (*tef1*) gene (Kopchinskiy et al., 2005) would be extremely expensive and time-consuming. Furthermore, the quantification of more than one strain by dilution plating is limited if one of the strains grows faster and completely covers the agar plate before all colonies could be counted (Dodd et al., 2004b).

Biochemical characterization of *Trichoderma* strains may be applicable for species identification or studying the diversity within and between certain species of the genus. Methods of isoenzyme analysis have been succesfully applied, e.g., for the differentiation between morphologically undistinguishable strains belonging to the *T. harzianum* species complex (Grondona et al., 1997), or for the characterization of winter wheat rhizosphere-derived *Trichoderma* strains belonging to various species based on their well-defined, characteristic isoenzyme patterns (Kredics et al., 2012). Metabolic profiling by BIOLOG Phenotype MicroArray on 95 carbon sources has also been applied to differentiate among *Trichoderma* strains at the species level (Hoyos-Carvajal et al., 2009), furthermore, the diversity of certain taxa, in particular the *T. harzianum* species complex, including isolates with antagonistic potential against the plant pathogenic fungus *Sclerotinia sclerotiorum*, was also found to be clearly reflected in their BIOLOG metabolic profiles (Lopes et al., 2012). However, although these approaches proved to be applicable to investigate the biochemical diversity between—and in some cases also within—distinct species of the genus *Trichoderma*, they are not suited well enough for monitoring purposes.

Although benomyl resistant mutants were succesfully used to examine the rhizosphere competence of *T. harzianum* strains in an early study (Ahmad and Baker, 1987), mutants might behave differently from the wild type strain in agricultural habitats, therefore methods enabling the monitoring of the wild type strain's population dynamics would be more preferable (Green and Jensen, 1995). The availability of the highly sensitive molecular biology tools summarized below, some of them being also highly specific, may fulfill the emerging need for high-throughput, rapid, and cheap monitoring techniques to detect beneficial or harmful strains and species from the genus *Trichoderma* in various agricultural environments.

## Immunological approaches

Immunological assays provide a range of possibilities for studying the growth dynamics of *Trichoderma* species during their antagonistic interactions with their saprotrophic target fungi in complex natural environments. Immunoassays as fast and sensitive detection methods were used to precisely identify and quantify *Trichoderma* species in soil or other complex environments. The introduction of hybridoma technology resulted in the exploration and application of specific monoclonal antibodies (MAbs) from animals (e.g., mice) to quantify *Trichoderma* biomass in soil. Enzyme-linked immunosorbent assays (ELISA) or immunofluorescence was employed to detect fungi in soil. MAbs of the immunoglobulin sub-class IgG_2a_ produced by the cell line *Th*.HD3^3^ from murine splenocytes could specifically recognize an antigen, a protein epitope which is part of a glycoprotein molecule from a number of *Trichoderma* species. This MAbs-based immunological assay has been used for the specific identification of living *T. harzianum* mycelia colonizing peat-bran medium (Carter and Lynch, 1991; Thornton et al., 1994).

More importantly, immunological assays can not only discriminate between mixed populations but also between different growth states of *Trichoderma* species. In the study of Thornton et al. (1994), a MAb-based ELISA was used to detect the active mycelium of *T. harzianum*. In contrast, murine MAbs produced from a hybridoma cell line, *GH5*, can only recognize antigens from phialoconidia of *T. harzianum* and *T. viride* isolates but not from chlamydospores or mycelium (Thornton and Dewey, 1996). MAbs-based assays that quantify the biomass of *Trichoderma* strains and detect their different components can be employed in the quantitative analysis of population dynamics of BCAs. MAbs can also be used to detect and quantify the growth of soil borne pathogens, such as *Rhizoctonia solani* and their growth dynamics under the influence of the hyperparasitic *T. harzianum* artificially introduced into composts (Thornton et al., 1999). The high level of specificity of the monoclonal antibody MF2 (raised against the constitutive glucoamylase enzyme) as a molecular marker for the recovery and detection of *Trichoderma* species has been demonstrated in the study of Thornton et al. (2002). MF2 specific for *Trichoderma* species and a group of their close relatives prepared from a commercial β-1,3-glucanase allowed the fast detection and visualization of *Trichoderma* species in soil or other complex matrices containing mixed fungal populations. The examined *Trichoderma* species have been shown to greatly inhibit the saprotrophic growth of *R. solani* in composts. By immunofluorescence micoscopy using MF2, *Trichoderma* isolate S-B2 was shown to grow toward and coil around the hyphae of the host in dual cultures of *Trichoderma* and *Rhizoctonia* (Thornton et al., 2002).

Immunological methods are also able to distinguish between rapid mycelial growth of *Trichoderma* and other components such as conidia and spores. In the study of Thornton (2004), the *Trichoderma*-specific monoclonal antibody MF2 was used to quantitatively assess the population dynamics of BCAs from the species *T. harzianum* and *T. asperellum* in antagonistic interaction with the pathogen *R. solani* in microcosm studies. A *R. solani*-specific monoclonal antibody (EH2) recognized an extracellular glycoprotein secreted from the growing tip of *Rhizoctonia* mycelia. Standard curves were constructed from extracts of lyophilized mycelium containing quantifiable and repeatable sources of the antigens. By using these curves, lyophilized mycelial biomass could be calculated, which allowed to comparatively estimate the mycelial growth of *Trichoderma* species and *R. solani*. *Trichoderma* species were able to prevent *R. solani* growth by competing with the pathogen for nutrients (Thornton, 2004).

The above studies indicate that the development of immunological assays allows the species-level detection and identification of *Trichoderma* strains, however, a shortage of these techniques is that serology is not specific at the isolate level.

## Introduction of exogenous marker genes

**Figure 1:**
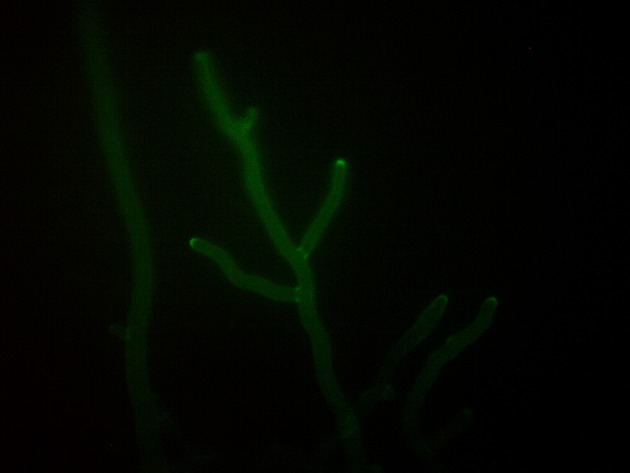
Hyphae of *Trichoderma pleuroti* expressing the green fluorescent protein (photo: Lóránt Hatvani).

Genetic engineering of BCAs with exogenous markers, e.g., the green fluorescent protein (*gfp*) (Figure 1), hygromycin B phosphotransferase (*hygB*), and/or β-glucuronidase (GUS) encoding genes is a powerful strategy to detect and monitor individual *Trichoderma* strains in agricultural habitats (Green and Jensen, 1995; Lo et al., 1998; Bae and Knudsen, 2000).

*Trichoderma harzianum* TF3, a propiconazole-resistant strain with the ability of survival at relatively high densities in the phylloplane of tomato and grapevine and high antagonistic potential against *Botrytis cinerea* on these crops was transformed to hygromycin B resistance by high-voltage electric pulse with the vector pHATα (a derivative of pAN7-1) containing the hygromycin B phosphotransferase (*hph*) gene of *E. coli*, which confers hygromycin B resistance as a selectable marker (Migheli et al., 1994). All transformants showed higher survival rates on tomato phylloplane than the wild type strain, all of them being mitotically stable after several passages in the absence of selective pressure and on tomato plants during 2 weeks, without losing the ability to grow in the presence of both hygromycin B and propiconazole. Thrane et al. (1995) used the polyethylen-glycol method to transform the same *T. harzianum* strain with both pNOM102 and pAN7-l carrying the *Escherichia coli* β-glucuronidase (GUS) and *hygB* resistance genes, respectively. The resulting transformants could be visualized under an epifluorescence microscope. Three methods were used to assay the GUS activity with 5-bromo-4-chloro-3-indolyl-β-D-glucuronide: the transformant strains were tested in a microtiter plate by incubating fresh mycelium, non-sporulating transformants were assayed for GUS activity on plates by wounding the mycelium, or the GUS activities of mycelial extracts were measured. Three of the transformants, *T. harzianum* T3a, T3b, and T3c proved to be mitotically stable for 8 months with respect to both genes. Green and Jensen (1995) studied the population activity and stability of the GUS-transformed *T. harzianum* strain T3a in the rhizosphere of sphagnum peat-grown cucumber plants. This was the first study using the GUS marker to monitor the presence, population dynamics and activities of a *T. harzianum* strain introduced into the rhizosphere. The authors proved the genetic stability of the transformant during its growth in a natural potting mixture, and observed a high GUS-production in conidia preparing for germination as well as during subsequent mycelial growth. The results demonstrate the applicability of a GUS-transformed strain to detect the presence and follow the population dynamics and activities of a specific *T. harzianum* strain in the environment. Bowen et al. (1996) transformed another *T. harzianum* strain, M1057, shown to be effective against *Sclerotium rolfsii*, with the plasmids pAN7-l and pNOM102. Two transformants proved to be mitotically stable and their growth rates were not different from the wild type. In order to evaluate the effect of transformation on biocontrol ability, the parental strain and the selected transformants were compared for their abilities to protect lentil (*Lens culinari*s) from the parasitic attack of *S. rolfsii*. Both the wild type and the transformant *T. harzianum* strains could rapidly colonize the soil with fast growth at the surface and inhibit *S. rolfsii* growth (Bowen et al., 1996).

To monitor the hyphal growth, activities and survival of a *T. harzianum* strain, Bae and Knudsen (2000) transformed strain ThzID1 with plasmids carrying the *gfp* (pTEFEGFP), GUS (pNOM102), and *hygB* (pAN7-2) genes. The mitotic stability of the cotransformants and their capacity to colonize the resting sclerotia of the plant pathogen *Sclerotinia sclerotiorum* in soil were investigated. The cotransformant ThzID1-M3 proved to be the only strain showing stable expression of all three introduced exogenous marker genes. In order to confirm the genomic integration of the introduced plasmid DNAs, the total genomic DNAs of ThzID1-M3 and the parental strain ThzID1 were tested by Southern hybridization with a pTEFEGFP fragment (*Hind*III-*Bam*HI), a pNOM102 fragment (*Nco*I-*Nco*I), and a pAN7-2 fragment (*Hind*III-*Eco*RI) to confirm the genomic integration of the introduced marker genes. According to the results of this study, cotransformation with GUS and *gfp* may provide a useful tool to detect and monitor specific *T. harzianum* strains released into the soil. ThzID1-M3 was the most succesful, colonizing ~60% of the sclerotia. A subsequent study examined, whether *Aphelenchoides* sp. (a fungivorous nematode) affects the growth and proliferation of ThzID1-M3 in soil (Bae and Knudsen, 2001). Soil treatment with a ThzID1-M3 formulation was found to stimulate the growth of fungivorous and other nematode populations, and a fungivorous nematode population could significantly reduce the colonization of *S. sclerotiorum* sclerotia by ThzID1-M3 in soil. Fungivorous nematodes migrate to soil locations with large quantities of available nutrients. Based on the results of the study, the *Trichoderma* strain introduced into the soil as a pellet formulation formed a hyphal net of high-density, which may be able to attract nematodes by serving as an appropriate nutrition source. Orr and Knudsen (2004) used the GUS- and *gfp-*labeled *T. harzianum* strain ThzID1-M3 to quantitatively measure the biomass changes of a fungal BCA in nonsterile soil, where the populations of indigenous bacteria and fungi interfere with traditional biomass determination procedures. The marker genes GUS and *gfp* allowed differentiation between the introduced BCA and the indigenous *Trichoderma* populations, and demonstrated that the monitoring of *gfp*-labeled *T. harzianum* by epifluorescence microscopy was a useful method for distinguishing active hyphal biomass functional for biological control from inactive chlamydospores and conidia enumerated by plate counts. Kim and Knudsen (2008) developed a set of specific PCR primers for ThzID1-M3 with high reproducibility and precision. Methods applying real-time PCR and epifluorescence microscopy-based image analysis were subsequently refined and evaluated in order to quantify the colonization dynamics of *S. sclerotiorum* sclerotia by ThzID1-M3 (Kim and Knudsen, 2011). In a recent study, Contina et al. (2017) hypothesized that the use of *T. harzianum* ThzID1-M3 would allow direct observation of the biocontrol mechanisms employed by strain ThzID1-M3 to reduce infection and reproduction of the potato cyst nematode *Globodera pallida*. Strain ThzID1-M3 proved to be useful to monitor the biocontrol interactions between *T. harzianum* and the nematode: the BCA significantly reduced *G. pallida* infection and reproduction in potato roots.

Lu et al. (2004) introduced the *gfp* gene into *T. atroviride* strain P1, which is able to suppress diseases caused by *Pythium ultimum* and *R. solani*. The authors proved that transformation with the *gfp* gene had no affect on the biocontrol activity of the *T. atroviride* P1 strain. Kowsari et al. (2014) transformed the *T. harzianum* strain ABRIICC T8-7MK, resulting from protoplast fusion, with the *amdS* and *gfp* genes, and selected a cotransformant which was subsequently tested against *R. solani*. The cotransformant was able to colonize potato tubers affected by black scurf and planted into agricultural soil treated with the *Trichoderma* strain. After 20 days of incubation, *Trichoderma* was found to completely colonize *R. solani* sclerotia and produce conidia totally covering the black scurf.

Although green fluorescent protein, hygromycin B resistance or GUS activity enable the development of simple tools for the monitoring of biocontrol strains (Bowen et al., 1996), the application of organisms genetically modified with exogenous markers as BCAs is a very tedious alternative due to the public concern about the release of GMOs into natural environments, which also needs specific permission in several countries including the European Union (Dodd et al., 2004b). Therefore, there is a constant need for the development of molecular tools based on endogenous DNA markers enabling the specific monitoring of distinct, genetically unmodified biocontrol *Trichoderma* strains in agricultural environments.

## Fingerprinting techniques

In general sense, fingerprinting techniques are DNA-based methods relying on PCR, digestion with restriction enzymes and/or Southern hybridization, generating banding patterns that can be compared with each other and allow the identification of characteristic, strain-specific DNA fragments.

The specific tool known as DNA fingerprinting was used by Schlick et al. (1994) to prove that different patented strains of *T. harzianum* and their gamma-ray induced mutants can be distinguished by using oligonucleotides (CAC)_5_, (GTG)_5_, GGCATCGGCC, and M13 sequence GAGGGTGGCGGTTCT as PCR primers and the wild-type M13 phage oligonucleotides (GTG)_5_ and (CT)_8_ as hybridization probes. The authors investigated *T. reesei* and strains of *T. harzianum* including LC-1 (a biocontrol strain), as well as LC-2 and LC-3 (its two irradiation mutant derivatives). As a result, discriminative fingerprint patterns were obtained, and all examined strains could be succesfully distinguished from each other.

The first technique that proved to be capable of distinguishing a commercially used biocontrol strain, *T. harzianum* T-39 used as a BCA against *B. cinerea*, from other *Trichoderma* strains was designed by Zimand et al. (1994). All examined *Trichoderma* strains could be distinguished based on their unique banding profiles generated by a set of 10 mer primers used in a random amplified polymorphic DNA (RAPD) procedure. This technique has some advantages when compared to morphology-based methods: it is less time-consuming and can be conducted using small amounts of DNA.

Instead of random sequences, Arisan-Atac et al. (1995) used wild-type M13 phage DNA GAGGTGGNGGNTCT, (GACA)_4_ and (GTG)_5_–previously used for RFLP fingerprinting—for the PCR generation of banding patterns, which was named RAPD fingerprinting. The authors analyzed 11 strains of *T. viride*, 2 strains of its teleomorph *Hypocrea rufa* and 9 isolates representing further *Trichoderma* species (*T. citrinoviride, T. parceramosum, T. harzianum, T. koningii, T. longibrachiatum, T. piluliferum, T. polysporum, T. reesei, T. saturnisporum*) which were screened for their antagonistic activities against *Cryphonectria parasitica*. The results showed that all three primers amplified 12–15 fragments characteristic to each strain.

To successfully differentiate the commercially used *T. harzianum* strain C65—a strain with biocontrol activity against *B. cinerea* causing kiwifruit stem-end rot—from other closely related *Trichoderma* species including isolates of *T. koningii, T. viride, T. reesei, T. atroviride*, and *T. aureoviride*. Bowen et al. (1996) designed a restriction fragment length polymorphism (RFLP) assay using the *Hind*III enzyme. Southern blot hybridisation probes for the RFLP analysis were generated by RAPD using the DNA of *T. harzianum* C65 as template. The method was capable to detect the BCA in the commonly isolated mycota. To facilitate the screening of a large number of samples, Dodd et al. (2004a) combined this isolate-specific RFLP marker with a dot-blot assay, which was used to monitor the spread and survival of *T. atroviride* C65 on leaves of kiwifruit both in shadehouse and in the orchard over two subsequent seasons. By this procedure, the authors have successfully proved that strain C65 is an ideal BCA, as it did not only survive but also spread to uninoculated trees and fruits, protecting them from post-harvest fruit rot.

Bulat et al. (1998) evaluated the ability of universally primed (UP) PCR and ITS-ribotyping to discriminate closely related *Trichoderma* and *Gliocladium* strains and to identify markers for fast and reliable detection assays. Several isolates of *T. harzianum, T. hamatum, T. koningii, T. polysporum, T. virens, T. viride*, and *G. roseum* were tested in a cross dot-blot hybridisation carried out using UP-PCR amplification products, and similarity among the strains was determined. The results suggested that the combination of UP-PCR and ITS-ribotyping techniques may become a valuable tool for intra-species distinction.

Forty-two isolates of *T. harzianum* and *T. viride* from Phillippine rice fields were analyzed by Cumagun et al. (2000) and characterized using rDNA-ITS1 analysis and UP-PCR with the 16–21 mer primers L45, 3-2, AA2M2, AS4, AS15, AS15inv, L21, Fok1, L15/AS19, and 0.3-1. Strains were characterized to the species level based on UP-PCR banding profiles and the length and *Sau*3A restriction patterns of the ribosomal DNA. The authors have concluded that UP-PCR combined with the analysis of rDNA-ITS1 is a useful tool for the identification of isolates that are indistinguishable on the basis of their morphology. The combination of UP-primers L21 and Fok1 could distinguish the promising biocontrol strain *T. harzianum* 94-016 from other *Trichoderma* isolates, therefore having the potential to be used for monitoring purposes.

Lübeck and Jensen (2002) combined dilution plating and UP-PCR with the above listed set of primers for monitoring the commercial biocontrol *Trichoderma* strains *T. harzianum* KRL-AG2 (Bio-Trek 22G), Supresivit (Supresivit), T39 (TRICHODEX), ATCC20476 (Binab T, type 1) and Promot 1 (Promot), *T. polysporum* ATCC20475 (Binab T, type 2), and *T. koningii* Promot 2 (Promot). Several primers generated characteristic banding patterns for each strain, allowing the commercially used strains to be differentiated from others. The results have shown that this method is suitable for monitoring the species composition in different glasshouses. The spread of the biocontrol strains could also be monitored, and the necessity of reintroduction could be easily detected.

Freeman et al. (2002) conducted arbitrarily primed PCR (ap-PCR) in an experiment to assess the use of the strains *T. harzianum* T39 (TRICHODEX) as well as T161 and T166 for controlling *B. cinerea* and *Colletotrichum acutatum* in strawberry, and to investigate the population survival of these 3 *T. harzianum* strains as well as *T. asperellum* T-105 on strawberry leaves. The primers (GACAC)_3_, (GACA)_4_, (TGTC)_4_, and (CAG)_5_ used for ap-PCR were derived from microsatellite or repeat sequences. The PCR banding patterns generated by ap-PCR proved to be suitable for grouping the isolates at the species level.

Fanti et al. (2003) evaluated fingerprinting with random, minisatellite and microsatellite primers for the environmental monitoring of two promising biocontrol *Trichoderma* strains effective against *Cytospora* canker of peach and found that the M13 minisatellite revealed unique fingerprints for both strains. Four months after the application to the soil under the canopy of peach trees, the antagonists could be recovered from above plant parts but not from soil, while after 1 year they were lost.

Naef et al. (2006) used a microsatellite-based method to determine the fungal biomass in decomposing plant material. PCR was carried out with fluorescent primers, which co-amplify fragments of different length from the reference and target strains. The primer pair MS-Ta4 generated a 132 or 142 bp long fragment from the investigated *Trichoderma* strains. *T. atroviride* P1 and *T. harzianum* RAC616 possess the alleles of 132 bp (13 TG repeats) and 142 bp (18 TG repeats), respectively. The fragments were separated by a capillary sequencer with fluorescent detection and the biomass of the fungi could be calculated by target/reference ratio of the fluorescent signal.

The RAPD-PCR method combined with benomyl resistance improved the identification of biocontrol strains from field samples. Tachapattaworakul et al. (2008) used this technique for monitoring of *T. atroviride* strains CBS 470.94, CBS 350.93, L2, and OB. The RAPD procedure with primer OPC8 generated a fragment of 3,000 bp specific to the biocontrol strain L2. This strain showed tenfold level of resistance to benomyl when compared to the other strains examined, which made its isolation from soil easier. The applied method allowed the recovery and identification of strain L2 from the soil 4 months after its inoculation.

Although the PCR-based fingerprinting tools represent simple and flexible methods, they are not always strain-specific and their reproducibility may be affected by the low annealing temperature and short primer size (Hermosa et al., 2001). However, strain-specific fragments generated by such techniques have the potential to be converted into monitoring markers based on SCARs (sequence characterized amplified regions) (Paran and Michelmore, 1993).

## Species-specific PCR

The use of specific primers is a powerful tool for distinguishing individual *Trichoderma* species in agricultural environments. Chen et al. (1999) developed a species-specific primer-based test to identify *T. harzianum* biotypes Th2 and Th4 (redescribed later as *T. aggressivum* f. *europaeum* and *T. aggressivum* f. *aggressivum*, Samuels et al., 2002), causing green mold diseases on *Agaricus bisporus* crops in Europe and North America, respectively. The specific primers were designed from the sequence of a 1,150 bp RAPD-PCR product generated by primer 232, which proved to be unique to *T. aggressivum*. The primer pair Th-F/Th-R (Table 1) gave no product from the DNA samples of *T. harzianum*, several biocontrol *Trichoderma* strains, or from 31 other genera and species of fungi. The method can be used efficiently for the detection of *T. aggressivum* in green mold-affected mushroom compost. Kredics et al. (2009) described a multiplex PCR assay allowing the detection of *T. pleuroticola* and *T. pleuroti* (formerly *T. pleurotum*) (Park et al., 2006), the green mold pathogens of oyster mushroom. Primers FPforw1, FPrev1, and PSrev1 (Table 1) were designed based on DNA sequences located within the 4th and 5th introns of the translation elongation factor 1 α (*tef1*) gene. By the use of the three-primer set in a multiplex PCR, a 447 bp fragment is amplified from both *T. pleuroti* and *T. pleuroticola*, while in the case of *T. pleuroti* an additional, 218 bp specific product is also formed. The primers showed no cross reaction with other *Trichoderma* species or species from other fungal genera. The technique proved to be suitable for the detection of *T. pleuroti* and *T. pleuroticola* directly from green mold-affected *Pleurotus* substrate without the need of strain isolation, furthermore, it could also be used to demonstrate the presence of *T. pleuroticola* in the growing substrates and on the surface of the fruiting bodies of wild oyster mushroom.

**Table 1:** Examples of species-specific PCR protocols for *Trichoderma*.

**Species specificity**	**Primer sequences (5^′^ to 3^′^)**	**PCR conditions**		**Product size**	**References**
[15pt]*T. aggressivum*	Th-F: CGGTGACATCTGAAAAGTCGTGTh-R: TGTCACCCGTTCGGATCATCCG	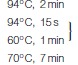	35 cycles	444 bp	Chen et al., 1999
*T. pleuroti* and *T. pleuroticola*	FPforw1: CACATTCAATTGTGCCCGACGA FPrev1: ACCTGTTAGCACCAGCGC	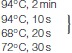	35 cycles	447 bp	Kredics et al., 2009
*T. pleuroti*	FPforw1: CACATTCAATTGTGCCCGACGA PSrev1: GCGACACAGAGCACGTTGAATC	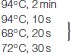	35 cycles	218 bp	
*T. harzianum*	HAR-1.6F: GTACCTCGCGAATGCATCTA HAR-1.6R: GGCTATGACCATGATTACGC	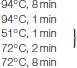	32 cycles	1,600 bp	Meena, 2009
*T. hamatum*	HAM-450F: TTGACACGGTTCTATAATTACCAA HAM-450R: TGACTTAAGTAAGCCGGGTCAAG	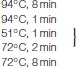	32 cycles	400 bp	
*T. harzianum*	2F2: TGGCTCGTCGTAGTTCGGAGAAG 2R2: CCAGATCGGCCACCGAAGAAAC	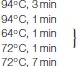	30 cycles	278 bp	Pérez et al., 2014
*T. harzianum*	2F2: TGGCTCGTCGTAGTTCGGAGAAG 2R3: GCCACCCACCGCGGGATTCA	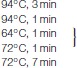	30 cycles	448 bp	
*T. harzianum*	2F2: TGGCTCGTCGTAGTTCGGAGAAG 2R2: CCAGATCGGCCACCGAAGAAAC	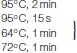	40 cycles	278 bp	
*T. harzianum*	ITS1 S: TACAACTCCCAAACCCAATGTGA ITS1 R: CCGTTGTTGAAAGTTTTGATTCATTT ITS1 TM Fam: FAM- AACTCTTATTGTATACCCCCTCGCGGGT-TMR	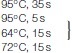	35 cycles	211 bp	López-Mondéjar et al., 2010
*T. virens*	TvCTT_56_f: CTTGATGACAAGCCAAAAGG TvCTT_56_r: GAAGAGAGGACATAGGGTCTGG	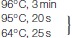	9 cycles(−0.5°C per cycle)	289 bp	Geistlinger et al., 2015
	TvCAT_32_f: GTGTAGCAGCCCAACAGTCC TvCAT_32_r: CAGGTGTCGTGACAGATTCG	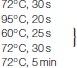	31 cycles	409 bp	
	TvCTTT_29_f GGAAGATAGCACGATGAAGTCG TvCTTT_29_r AACCGTGGAAGTAGGTGTCG			350 bp	
	TvCTTTT_27_f: TCATCCACCCTGCTAACTCG TvCTTTT_27_r: CGCTGCGTCATCCTAAACC			420 bp	
	TvAAC_21_f: CACCATTCCATTATTACGCGACG TvAAC_21_r: CTGCACTCCCTCCCAATGC			234 bp	
	TvCAG_13_f: CCCAGGAAACCCTCAGAACG TvCAG_13_r: TCTTTGCAGTTTCCAAGTCGG			180 bp	
	TvGAAA_34_f: GGGGTGCTGAATAGCTAACG TvGAAA_34_r: TGCCGTCTTGTCTTATTTTCG			325 bp	
	TvTGTC_18_f: GTGGTGAGGACTTGCTTGG TvTGTC_18_r: TCTGCCTGTCAGTTGTTTGC			425 bp	
	TvGAT_18_f: GGGATCTGATTTGGCCTACC TvGAT_18_r: ACTTCCCCCATCCAATAACG			371 bp	
	TvCA_39_f: GCATCTGCACCTGATATATTCC TvCA_39_r: CCTTGTACGATCTCCAGAACC			256 bp	
	TvGTT_23_f: GCATCAAAGCGTGCTGTTGG TvGTT_23_r: GCAAACACAAGCTGACAATGC			216 bp	
	TvAG_29_f: TGTGCCCACTGAGATTTCG TvAG_29_r: TCAGCATGAGATTACACATACCG			449 bp	
*T. atroviride*	Q01_4F: GCACACCAACTGCTGGAGCTT Q01_4R: CACGCTGACAATGACCGACAC	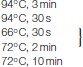	27 cycles	1,017 bp	Skoneczny et al., 2015
*T. atroviride*	X18_3F: AGGCACAGTCCCCTGTTTAGT X18_5R: TGACGATCCTGGTAAGGTTTG	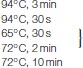	35 cycles	358 bp	
*T. atroviride*	Z04_2F: TTACCCAGTGCGGAATCCAAA Z04_2R: TATACGGCGCCTTCCACATTG	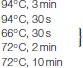	27 cycles	1,450 bp	
*T. atroviride*	Q01_3F: AAGCAAGGGGGTTGGCAAGTA Q01_3R: GAGAAGGGGTTCCCTGCAGAA	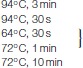	35 cycles	760 bp	Oskiera et al., 2017
*T. atroviride*	X18_1F: GACTAGGTGGTCACAGACGAAAX 18_3R: GGAAACTCCATCACAAATCCA	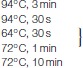	35 cycles	630 bp	
*T. harzianum*	QTh_5F: GGGTTGTTCGGATGGAAG QTh_4R: GTTGGAGATGGGAGGAAGA	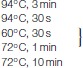	35 cycles	2,000 bp	

Meena (2009) described a technique for the detecion of the species *T. harzianum* and *T. hamatum*, known as potential biocontrol agents of different plant pathogenic fungi. The author designed SCAR primers (Table 1) based on the sequence of species-specific RAPD fragments. A similar method was developed by Pérez et al. (2014) (Table 1), which enables monitoring the growth and colonization of *T*. cf. *harzianum* in experimental communities, however, due to the cosmopolitan and diverse nature of the species the authors did not suggest the use of this tool for assessing the presence of *T*. cf. *harzianum* in complex environmental samples such as soil.

In order to monitor and quantify *T. harzianum*, López-Mondéjar et al. (2010) designed a primer set and a TaqMan probe for the ITS region (Table 1). The quantity of ITS copies was calculated based on real-time PCR results and a correlation of 0.76 was obtained between ITS copy number and the fungal biomass determined by optical microscopy and image analysis. The authors suggest that quantification of fungi in soil samples can be performed precisely using real-time PCR data. The primers and the probe were designed for pure cultures of *T. harzianum*, however, they might be applied for the detection and quantification of the fungus in soils and other organic materials. This method was successfully used by Beaulieu et al. (2011) to monitor the population of *T. harzianum* in peat and green compost.

Geistlinger et al. (2015) introduced a tool based on the use of simple sequence repeats (SSRs) using Touchdown PCR to track and quantify the species *T. virens*. Primers were designed for 12 different loci. The results confirmed the root-endophytic lifestyle of this species in tomato plants: the fungal biomass in the plant tissues was quantified and co-colonization of the roots by different strains of *T. virens* could also be observed.

Skoneczny et al. (2015) examined the genetic diversity of environmental *T. atroviride* isolates using cleaved amplified polymorphic sequence (CAPS) markers. Following the amplification of RAPD regions by PCR primers (Table 1), the obtained amplicons were digested with defined restriction enzymes (*Bsl*I, *Dra*I, *Taq*I). Three CAPS markers were found to distinguish *T. atroviride* from other species; therefore they have the potential to be used for the identification and monitoring of *T. atroviride*, particularly in environmental specimens.

Recently, Oskiera et al. (2017) described a multiplex PCR assay for monitoring the population levels of *T. atroviride* and *T. harzianum* applied as biopreparations in the field. For the detection in soil, a maximum of 3 primer pairs targeting markers TaQ (Q01_3F/Q01_3R) or TaX (X18_1F/X18_3R) (Table 1), the *Trichoderma* chitinase-specific chit42_1af/chit42_2ar (Kullnig-Gradinger et al., 2002) and ITS primers (5.8SR/LR6) specific to fungi were combined for the detection of *T. atroviride*, while two markers, TVD (TVD_If/TVD_Ir) (Bhat, 2007) and ThQ (QTh_5F/QTh_4R) (Table 1) were combined in multiplex-PCR for *T. harzianum* detection. Primers targeting the β-tubulin gene of *Trichoderma* spp. (β-tub-F, β-tub-R) (Chen et al., 1999) served as a control. The results of the study confirmed the increased presence of *T. atroviride* and *T. harzianum* in the soil even 2 years after the application of biopreparations, while these species were not found in untreated soil samples (Oskiera et al., 2017).

## Design of strain-specific PCR primers

Although the above mentioned species- and genus-specific detection tools are very valuable for studying the population dynamics or epidemiology of certain *Trichoderma* species, due to the lack of strain specificity they are not the most reliable options for the environmental monitoring of particular biocontrol strains.

Nucleic acid-based endogenous markers are unique DNA sequences distinguishing a particular strain from all others. Such markers have the potential to be exploited in highly specific and sensitivite PCR-based assays. SCARs based on strain-specific fragments generated by fingerprinting methods allow the development of conventional and real-time PCR-based strategies for the differentiation of a particular biocontrol *Trichoderma* strain from other strains of *Trichoderma* present in the field environment, even from those belonging to the same species.

The first strain-specific SCAR marker-based tool of *Trichoderma* monitoring was developed by Abbasi et al (1999) for the precise detection and monitoring of *T. hamatum* strain 382, a BCA effective in compost-amended substrates. The authors performed RAPD analysis and identified 3 diagnostic fragments of 0.35, 0.6, and 0.65 kb in the patterns generated by primers OPE-16, OPH-19, and OPH20, respectively. The fragments were cloned into vector, sequenced, and primer pairs were designed for each of them (Table 2). The resulting 3 SCAR primer-pairs could be successfully used to indicate the presence of *T. hamatum* 382 in 9 different compost, soil and potting mix samples fortified with the BCA, while 274 *Trichoderma* isolates deriving from 9 non-fortified samples gave negative results. The method proved to be applicable also for the detection of the SCAR fragments from crude DNA preparations extracted directly from compost-amended substrates.

**Table 2:** PCR-based tools developed for the strain-specific monitoring of *Trichoderma* strains.

**Strain specificity**	**Primer sequences (5^′^ to 3^′^)**	**PCR conditions**		**Product size**	**References**
*T. hamatum* 382	OPH19-F: CTGACCAGCCTGTTAAAATCAT OPH19-R: CTGACCAGCCCAAAGACTCCC	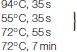	30 cycles	588 bp	Abbasi et al, 1999
	OPE16-F: GGTGACTGTGGCCTTGTTTGCATA OPE16-R: GGTGACTGTGAATGGCGAAGCTAC			347 bp	
	OPH20-F: GGGAGACATCGCATCTGCATGTAA OPH20-R: GGGAGACATCAACGATGATTCAGC			624 bp	
*T. atroviride* 11	11-A1: GGAAGCTTGGCGTTTATTGTACAA 11-A1c: GGAAGCTTGGGTATTGAGCTGGGC	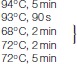	30 cycles	990 bp	Hermosa et al., 2001
*T. harzianum* 2413	BR1: TGAAGAGCGCCTCGACGA BR2: GGGTGATGATTTGCTGGC	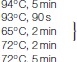	30 cycles	837 bp	Rubio et al., 2005
	Q2413f: TGGCGTTGAATTGAGTTTGTGT Q2413r: CCCTCCGTATGGGTTTTAAGGT	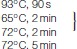	30 cycles	72 bp	
*T. atroviride* T1	PF74: GCAGGTCAAAGGCTAAAACG PR88: GCAAGAGGTTGATGGCAGTT	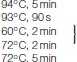	30 cycles	141 bp	Cordier et al., 2007
*T. virens* GV4	F116: GTACCTAGCTAACTGGGGTGCTG R331: ATAACCAGGCCGCGGCATCGGA	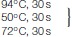	25 cycles	346 bp	Dodd et al., 2004b
*Hypocrea parapilulifera* Tp039	TpX021F2: GGTGTGGACAAGGGCGATATCTGA TpX021R: TTCCGCCACCCCCCATGTCCA	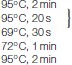	25-30 cycles	637 bp	Feng et al., 2011
*T. atroviride* Ta040	TaX131F: ACGGGAGCAATCTAAATACACCTA TaX131R: ACGGGAGCAATATGTCACGTT	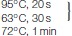	25-30 cycles	2,029 bp	
*H. parapilulifera* Tp039 and *T. atroviride* Ta40	TpX021F: TTCCGCCACCCAAGGAGTAAGCAG TpX021R: TTCCGCCACCCCCCATGTCCA	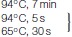	40 cycles	160 bp	
	TaX131Fnew: ACGGGAGCAATCTAAATACACCTAGCGACA TaX131Rnew: GCATTGTCCTTTTGGAGACTGTTGCCGA TpX021-FAM: [FAM]CAGGCAACGGGCGTAGGTGAAAAG[BHQ] TaX131-Cy5: [Cy5]CCTGCCGAGACTGGCCAATCTTTAAAG[BHQ]			157 bp	
*T. harzianum* AS12-2	15invFL1: CTGTGCTCCAATTGATCGACGA 15invRL1: GACAACTTGAAGGTAGACGAATCGTC 15invF2: TTGCTGGCGAATCGGAGGATAC 15invRL1: GACAACTTGAAGGTAGACGAATCGTC	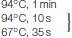 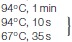	35 cycles 35 cycles	796 bp 627 bp	Naeimi et al., 2011
*T. virens* GvT6	TCGATCGGCACAGGCGATCG TGACGCCCGCAAGGTCGGTG	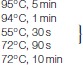	40 cycles	1 kbp	Baek and Kenerley, 1998
*T. atroviride* SC1	Ech42 Fw: GTTCTGAGGCTGGAAGTTGC Ech42 Rv: ACGCCGTCTACTTCACCAAC Ech42 P: 6FAMTACCCCTTCAATCACCAATTGTTAG-TAMRA	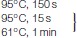	40 cycles	112 bp	Savazzini et al., 2008
*T. harzianum* T22	TH179F1: TTGCTGAATCTGTCCGAACC TH305R1: AACCTGCCTTCGTTTGGAG PrT22: [FAM]CCTTGGCGACCTTTTCACCAACAAGTC[BHQ]	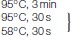	40 cycles	126 bp	Horn et al., 2016

The same strategy was applied by Hermosa et al. (2001), who aimed to develop a PCR technique for the monitoring of the patented *T. atroviride* 11 strain showing biocontrol activity against plant pathogenic fungi including *Acremonium cucurbitacearum, Aphanomyces cochlioides, Phoma betae*, and *R. solani*. RAPD analysis with primer OPF10 revealed two fragments (11-A1: 996 bp, 11-A2: 361 bp) from the DNA of *T. atroviride* 11, which were absent from the closely related *T. atroviride* 260 strain. The fragments were cloned, sequenced and subjected to nucleotide and protein BLAST analysis, which revealed no significant homology. Although primers 11-A1 and 11-A1c (Table 2) designed based on the sequence of the SCAR 11-A1 fragment proved to distinguish strain *T. atroviride* 11 from 26 other *Trichoderma* strains belonging to 7 species (Hermosa et al., 2001), later it turned out that they were not strain-specific as they also yielded the PCR product from 5 of the 9 *T. atroviride* strains examined by Cordier et al. (2007). In a subsequent study (Rubio et al., 2005), the same primer pair was tested against the DNA of further 27 *Trichoderma* strains, and it was also found to amplify a larger, 1.5 kb fragment from *T. harzianum* strain 2413. This fragment was cloned, and the primers BR1 and BR2 (Table 2), amplifying a 837 bp fragment from *T. harzianum* 2413 were designed based on the sequence information of the SCAR-fragment. The primers were tested on the DNA from 27 *Trichoderma* strains and 22 untreated soils, and proved to be specific to *T. harzianum* 2413. In addition, primers Q2413f and Q2413r (Table 2) along with a TaqMan fluorogenic probe were also designed based on the sequence of the 837 bp SCAR marker, in order to improve the sensitivity of the monitoring tool by the application of real-time PCR. The developed assay was able to quantify the DNA of the BCA from sterile soil fortified with 10^8^ conidia/g of soil but failed to detect the strain from a *Trichoderma* mixture introduced into the soil (Rubio et al., 2005). In a similar study, Cordier et al. (2007) developed a SCAR-based real-time PCR to specifically identify and study the population dynamics of *T. atroviride* strain T1. The authors of this study also applied the primers 11-A1 and 11-A1c to generate a fragment from strain T1, which was converted to a SCAR marker by sequence analysis. Among the 3-primer sets designed for the SCAR fragment, the primer pair PF74/PR88 (Table 2) generated the expected amplicon of 141 bp only from strains T1 and 1571. RAPD and AFLP (amplified fragment length polymorphism) fingerprints have shown that these two strains are identical for all the molecular characters tested and are therefore probably the parallel deposits of the same isolate. The same primer pair was used to develop a real-time PCR assay with SYBR Green detection, which proved to be applicable for monitoring the population dynamics of strain T1 in complex soil environments: detection of the BCA was possible at levels of 10^3^ CFU/g of nonsterile soil containing autochthonous *Trichoderma* populations (Cordier et al., 2007).

Dodd et al. (2004b) also used RAPD, and with primer OPAW04 they could identify a fragment unique to *T. virens* GV4, a strain with biocontrol abilities against both the vegetable pathogen *S. sclerotiorum* and the onion white rot pathogen *Sclerotium cepivorum*. Among the primers designed based on the sequence of the fragment, the primer pair F116/R331 (Table 2) gave the most consistent results during tests with further 5 *T. virens* isolates. The test was developed further to a duplex PCR system by including ITS4 and ITS5 (White et al., 1990), two universal primers targeting the ITS region as positive control to detect amplifiable fungal DNA, and was able to detect strain GV4 down to a concentration of 10 spores per gram nonsterile agricultural soil (Dodd et al., 2004b).

To quantify the presence of the biocontrol strains *Hypocrea parapilulifera* Tp039 and *T. atroviride* Ta040, Feng et al. (2011) developed primer pairs for strain-specific SCAR markers identified by RAPD primers OPX02 for Tp039 and OPX13 for Ta040. The primer pairs TpX021F/TpX021R and TaX131R/TaX131R amplified a 637 and a 2,029 bp fragment from strains Tp039 and Ta040, respectively (Table 2). Additional primers replacing the original forward or reverse primer were also designed. The TpX021F2/TpX021R combination amplifying a 160 bp fragment and the TaX131F/TaX131R2 combination forming a 157 bp fragment, discriminated Ta040 and Tp09 from all other strains tested. Fluorophore-labeled probes distinguishing the two strains were also designed and succesfully used in combination with the SCAR primers in multiplex real-time PCR for the examination of artificially inoculated soils as well as samples derived from a golf green which has been treated with a product containing both strains. The results revealed that the two biocontrol strains were not able to establish on the golf green and its surrounding area (Feng et al., 2011).

Instead of RAPD, Naeimi et al. (2011) performed UP-PCR with 5 universal primers both separately, and in pairwise combinations in order to search for endogenous markers specific to *T. harzianum* AS12-2, a strain with the potential to control the sheath blight disease of rice caused by *R. solani*. UP primers are primarily targeting more variable intergenic regions of the genome, therefore, this technique is especially useful in detecting intraspecific variations (Bulat et al., 1998). The application of UP primers AS15inv, AA2M2, as well as their pairwise combinations resulted in the amplification of unique fragments (905, 684, and 842 bp, respectively) from the genomic DNA of the *T. harzianum* AS12-2 strain. The sequences of the cloned fragments were used to design 14 oligonucleotide primers. Two primer pairs, 15invFL1/15invRL1 and 15invF2/15invRL1 (Table 2), designed from the sequence of the 905 bp SCAR marker, amplified fragments of the expected size (796 and 627 bp, respectively) from the DNA of T. *harzianum* AS12-2, but not from the DNA of further 111 *T. harzianum* isolates tested or of 84 further strains belonging to 5 other *Trichoderma* species occurring in rice fields (Naeimi et al., 2011).

Specific PCR markers for the monitoring of particular strains of a species can also be developed from sequences with known functions. In genetically modified strains, the sequences of the introduced genes may serve as targets of primer development. Baek and Kenerley (1998) developed a quantitative PCR method for the detection and quantitation of the *T. virens* strain GvT6, which carries the bacterial organophosphate degrading (*opd*) gene, therefore being potentially applicable for the bioremediation of soils contaminated with organophosphate pesticides. The assay based on the amplification of an *opd* fragment with *opd*-specific primers can be quantified by the application of a DNA standard containing the 1.5 kb *gpd* gene with 20 bp *opd* primer-binding sequences at both ends. The detection limit of the PCR assay from soil samples was 10–1,000 times lower than that of traditional dilution plating (Baek and Kenerley, 1998). The same Q-PCR method was used by Weaver et al. (2005) for a genetically modified derivative of *T. virens* Gv29-8, constructed by transformation with plasmid pCL1 carrying the *hygB* and the *opd* genes. The authors evaluated the genetic stability and ecological persistence of the strain over 243 days in soil mesocosms and found that both the genetically modified and the wild type strain were able to rapidly and efficiently colonize freshly added substrates even after the long-term incubation, indicating a high level of responsiveness (Weaver et al., 2005). A similar monitoring tool has been developed for studying the environmental fate of *T. reesei* strain QM6A, an efficient cellulase producer of biotechnological significance (Providenti et al., 2004). The strain was transformed with the hygromycin B resistance gene and specifically monitored in intact soil-core microcosms with primers targeting the introduced marker. Results of this study indicated that *T. reesei* may persist for at least 2 seasons in soil followed by its release from fermentation plants as spent biomass.

The increasing availability of *Trichoderma* sequences with known functions allows the identification of single-nucleotide polymorphisms applicable for the design of strain-specific monitoring tools. Savazzini et al. (2008) used the *ech42* endochitinase gene sequence to monitor *T. atroviride* strain SC1, a BCA against root rot pathogens, primarily *Armillaria* species in soils of orchards and vineyards, for which the development of RAPD-based SCAR markers was unsuccessful. The authors amplified the target gene from the BCA with primers Ech42 Fw and Ech42 Rv (Table 2), and compared its sequence with 34 other *ech42* sequences, which was followed by the design of the TaqMan probe Ech42 P (Table 2) targeting 2 nucleotide mutations within the first intron of the *ech42* gene of strain SC1. A second primer set and TaqMan probe to a common sequence of *tga3*, a gene encoding the G protein α subunit, were also designed to verify the presence of *Trichoderma* DNA in specificity tests and microcosm experiments. The detection and quantification limits of the resulting low throughput time, real-time PCR procedure were 6,000 and 20,000 haploid genome copies per gram of soil, suggesting that the method could be useful to monitor the fate of *T. atroviride* SC1 applied as an open-field BCA (Savazzini et al., 2008). This monitoring tool was incorporated into an intact soil microcosm technique developed to evaluate both the survival and the vertical dispersal of the SC1 strain (Longa and Pertot, 2009). Another study applying this monitoring tool revealed that strain SC1 tolerated both low temperature values during winter and fluctuations of humidity in soil after applications in a vineyard, could move beyond the treated soil area, migrated passively to the lower leaves of grapevine and became an integral part of the local microbiota (Longa et al., 2009).

Horn et al. (2016) performed genome mining to identify putative, new regions enabling the differentiation of various *T. harzianum* strains, and subsequently developed a multiplex Q-PCR assay for the monitoring of *T. harzianum* strain T22, an efficient BCA against *Calonectria pauciramosa*. The region upstream the coding part of the L-amino acid oxidase gene *aox1* proved to have sufficient genetic variation to discriminate between different strains of *T. harzianum* including T22, therefore a primer pair was designed for this region along with TaqMan probes specific to particular *T. harzianum* strains including T22 (Table 2). Strain T22 could be successfully monitored by the developed Q-PCR assay on the roots of greenhouse-cultivated tomato plants inoculated with the BCA (Horn et al., 2016).

The main problem during the use of DNA-based, strain-specific monitoring tools for the direct tracking of *Trichoderma* strains in agricultural environments is the difficulty in reproducing the results, which may be due to the presence of PCR inhibitors, such as humic acids and tannins—co-extracted with nucleic acids in the case of soil environments—and the presence of large amounts of DNA from other microorganisms in the examined habitats (Dodd et al., 2004b; Cordier et al., 2007). The inhibition of PCR in combination with the varying efficiency of DNA extraction between conidia and mycelia (Fredricks et al., 2005) may lead to an underestimation (Cordier et al., 2007), while the lack of discrimination between DNA from living and dead cells may result in an overestimation of the populations present (Pujol et al., 2006). It cannot be excluded either that a SCAR marker may be lost during field trials, however, this problem can be circumvented by the development and application of several independent strain-specific markers for the monitoring of a single strain (Hermosa et al., 2001).

## “OMICS” approaches in the service of *Trichoderma* monitoring

Since the release of the first complete *Trichoderma* genome sequence in 2008 belonging to the industrially important cellulase producer *T. reesei* (Martinez et al., 2008) and its subsequent comparative analysis with the genomes of the biocontrol species *T. virens* and *T. atroviride* (Kubicek et al., 2011; Schmoll et al, 2016), the number of full genome sequences publicly available in databases for *Trichoderma* strains is growing rapidly (Baroncelli et al., 2015, 2016; Kuo et al., 2015; Shi-Kunne et al., 2015; Compant et al., 2017), which opens the way for using comparative genome analysis to identify specific endogenous markers. An example is the study of Mahfooz et al. (2016), who performed a comparative *in silico* analysis of SSR sequences in the genomes of *T. reesei* and three biocontrol species of the genus, and developed 6-6 primer pairs for the amplification of polymorphic markers from *T. atroviride* and *T. harzianum*. In another study, Lange et al. (2016) compared the genome sequence of the remarkably effective biocontrol agent *T*. cf*. atroviride* strain LU132 with that of the less efficient strain *T*. cf. *atroviride* LU140 and identified a single functional nucleotide polymorphism (SNP), which proved useful for the development of an RFLP technique applicable for the differentiation of the two strains from each other.

The approach of full metagenome sequencing in the case of samples from agricultural habitats has the potential to reveal important information not just about *Trichoderma* but the entire microbiome, however, it requires sequencing platforms providing a very high coverage as well as substantial bioinformatics capacities. An alternate approach is metabarcoding, which may also provide valuable tools for the investigation of *Trichoderma* populations in agricultural and natural soil systems. Hagn et al. (2007) developed a set of primers targeting the ITS region for the cultivation independent detection and monitoring of *Trichoderma* species. The analysis of a clone library from PCR products amplified from arable soil revealed that *Trichoderma* clades comprising many species of biocontrol relevance were covered by amplification with the designed primer set. For *Trichoderma* diversity analysis, Meincke et al. (2010) developed another *Trichoderma*-specific primer pair targeting the ITS region and a semi-nested PCR to amplify fragments suitable for denaturing gradient gel electrophoresis (DGGE), which was tested on *Trichoderma* communities in the rhizosphere of various potato genotypes. However, the reverse primer used in this study binds to a still polymorphic region of ITS2, making several species undetectable, therefore Friedl and Druzhinina (2012) worked out an ITS-based metabarcoding approach based on six reverse primers used in combination with the forward primer ITS5, that amplify the entire diagnostic ITS1 and 2 region from all representatives of the genus. This culture independent PCR-based tool was succesfully used to reveal the restricted diversity of the genus *Trichoderma* in temperate soil of a riparian forest.

While PCR methods targeting genomic DNA amplify products from both active (e.g., growing hyphae) and inactive fungal elements (e.g., dead mycelia and dormant conidia), which may result in the overestimation of *Trichoderma* populations, transcriptomic approaches by the isolation of total RNA, its reverse transcription and the subsequent detection of cDNA have the advantage of allowing the detection of active *Trichoderma* populations. The previously mentioned method of López-Mondéjar et al. (2010) detecting genomic ITS sequences was further developed to a quantitative reverse transcribed PCR (qRT-PCR) tool by Beaulieu et al. (2011) to monitor the active population of *T. harzianum* in peat and green compost. The results of this study revealed a significant correlation between qRT-PCR and the parallelly applied serial dilution technique. In a more recent study, Mendoza-Mendoza et al. (2015) identified key genes involved in the morphogenesis of *Trichoderma* sp. “*atroviride* type B” and developed molecular marker-based assays to follow the development of the strain in soil environment. The authors developed an efficient total RNA isolation method from soil and followed the expression profiles of *Trichoderma* genes specific to different developmental stages (dormant conidia, germination stage, and vegetative hyphae) by qRT-PCR. Microarray technology offers further perspectives for studying *Trichoderma* populations in natural environments by following the expression changes of several genes. With the availability of field-portable microarray analysis systems (Chandler et al., 2010), this approach not only enables the monitoring of active *Trichoderma* populations, but may also provide information about the structure and dynamics of the entire microbial community, as well as about the physiological status of *Trichoderma* populations in the soil environment. For the selection of target genes appropriate for monitoring by microarrays, the results deriving from transcriptomic studies and comparative transcriptome analyses performed *in vitro* or in soil microcosm systems for *Trichoderma*-fungus (Atanasova et al., 2013; Steindorff et al., 2014; Morán-Diez et al., 2015; Perazzolli et al., 2016; Shaw et al., 2016) and *Trichoderma*-plant (Chacón et al., 2007; Samolski et al., 2009; Rubio et al., 2012; Shaw et al., 2016) interactions are highly valuable. Metatranscriptome analysis of agricultural habitats after the introduction of *Trichoderma* as a biocontrol agent has further potential to reveal important information about the changes of the entire, active microbiome, however, similarly to the sequencing of full metagenome, this approach is also expensive and time-consuming due to the necessity of appropriate, high-throughput sequencing and computational platforms, furthermore, the choice of an appropriate RNA isolation method is crucial due to the RNA's high susceptibility to degradation by RNase enzymes.

Besides substantially aiding the process of *Trichoderma* strain selection as well as the development of application strategies in the case of BCAs, the increasing amount of data available in the literature about the proteome, metabolome, and secretome of *Trichoderma* strains from different species (Sharma et al., 2009; Lorito et al., 2010; Druzhinina et al., 2012; Contreras-Cornejo et al, 2016) may also contribute to the development of new monitoring strategies based on the detection of specific cellular or secreted proteins or secondary metabolites. For example, the non-ribosomally synthesized peptaibols have the potential to form the basis of mass spectrometry-based, species-specific monitoring strategies, as particular *Trichoderma* species produce a unique peptaibiome which is different even from that of closely related species (Marik et al., 2017).

## Conclusions

During the past decades, the methods for monitoring *Trichoderma* populations in agricultural environments evolved from genus-specific, classical microbiological tools through species-specific immunological and fingerprinting techniques to high throughput, strain-specific real-time PCR methodologies. Table 3 shows a critical evaluation of the reviewed methods available for *Trichoderma* monitoring (detection and quantification) in agricultural environments, with special focus on their strengths and limitations. An ideal tool for monitoring *Trichoderma* species/strains in agricultural environments is expected to be cheap, highly sensitive, and reproducible, specific at strain (or at least at species) level, able to accurately quantify fungal densities by detecting only the active fungal elements, applicable to commercial BCAs, as well as easy to perform, without the need of extensive knowledge of classical taxonomy or expensive equipments. Although none of the discussed methods meet all these requirements, certain tools like the application of qRT PCR, strain-specific primers developed based on SCAR markers or comparative omics approaches possess a wide scale of advantages, making them suitable as parts of strategies aimed at *Trichoderma* monitoring in agricultural environments. Furthermore, it can be expected that the era of bioinformatically supported genomic, transcriptomic and metabolomic approaches will further increase our understanding of the population dynamics and ecology of *Trichoderma* in agricultural environments, thereby delivering useful information for the optimization of application strategies in the case of BCAs, and also supporting the development of adequate and efficient control methods for the harmful members of the genus.

**Table 3:** Critical evaluation of the advantages and limitations of techniques applicable for monitoring (detection and quantification) *Trichoderma* species/strains in agricultural environments.

**Monitoring method**	**Rapid—time consuming (1–5)**	**Cheap—expensive (1–5)**	**Culture-independent**	**Highly sensitive**	**Highly reproducible**	**Specificity (strain level: 1, species-level: 2, genus-level: 3)**	**Detects only active fungal elements**	**Provides information about populations of other microorganisms**	**Provides information about the developmental stage of the target fungus**	**World-wide applicable to commercial BCAs**	**No need of extensive knowledge of classical taxonomy**	**No need of expensive equipment**	**No need of extensive bioinformatic capacity**
**CLASSICAL TOOLS**													
Dilution plating on *Trichoderma*-selective medium for quantification	5	2	–	–	–	3	+	–	–	+	+	+	+
Culture-based morphological approaches for identification	5	1	–	–	–	2	+	–	–	+	–	+/–	+
**BIOCHEMICAL TOOLS**													
Isoenzyme analysis	4	3	–	–	–	2	+	–	–	+	–	+	+
BIOLOG Phenotype MicroArrays (metabolic profile analysis)	4	4	–	+/–	+/–	2	+	–	–	+	+	–	+
**MOLECULAR BIOLOGY TOOLS**													
Immunological assays based on monoclonal antibodies (ELISA, immunofluorescence)	2	3	+	+	+	2	+	–	–	+	+	–	+
Application of strains transformed with exogenous genetic markers (*gfp, hygB, GUS*)	2	1	+	+	+	1	+	–	–	–	+	+/–	+
DNA fingerprinting with oligonucleotides as PCR primers and/or hybridization probes	3	2	–	–	–	1–2	–	–	–	+	+	+	+
RAPD	1	2	–	–	–	1–2	–	–	–	+	+	+	+
RFLP with Southern blot	3	2	–	–	–	1–2	–	–	–	+	+	+	+
UP-PCR with ITS ribotyping	3	2	–	–	–	1–2	–	–	–	+	+	+	+
Application of CAPS markers (RAPD followed by digestion with restriction enzymes)	3	2	–	–	–	1–2	–	–	–	+	+	+	+
Classical PCR with specific primers	1	2	+	–	+	1–2	–	–	–	+	+	+	+
qPCR with specific primers	1	3	+	+	+	1–2	–	–	–	+	+	–	+
qRT-PCR	3	3	+	+	+	2	+	–	+/–	+	+	–	+
ITS-based metabarcoding	4	4	+	+/–	+/–	2–3	–	+	–	+	+	–	–
Full metagenome analysis	5	5	+	+/–	+/–	2	–	+	–	+	+	–	–
Full metatranscriptome analysis	5	5	+	+/–	+/–	2	+	+	+	+	+	–	–
Analytical detection of specific metabolites	4	4	+	+/–	+/–	2–3	+	–	–	+	+	–	–

*+, tool meets the requirement; −, tool does not meet the requirement; +/-, meeting the requirement depends on the specific method/equipment used*.

## Author contributions

LC and CV reviewed the immunological approaches. OK and JK reviewed the introduction of exogenous marker genes. RB and NA reviewed fingerprinting techniques. LH and HA reviewed species-specific PCR and metagenomics tools. VN and LK reviewed the design of strain-specific PCR primers. LK and CV prepared the abstract, introduction and conclusions, and assembled the manuscript. Critical reviewing of all content and editing of the manuscript were performed by LK, CV, JK, NA, and LH.

### Conflict of interest statement

The authors declare that the research was conducted in the absence of any commercial or financial relationships that could be construed as a potential conflict of interest. The reviewer FV and handling editor declared their shared affiliation at time of review.
